# Experimental evolution under hyper-promiscuity in *Drosophila melanogaster*

**DOI:** 10.1186/s12862-016-0699-8

**Published:** 2016-06-16

**Authors:** Jennifer C. Perry, Richa Joag, David J. Hosken, Nina Wedell, Jacek Radwan, Stuart Wigby

**Affiliations:** Edward Grey Institute, Department of Zoology, University of Oxford, Oxford, UK; Jesus College, University of Oxford, Oxford, UK; University of Exeter, Penryn, UK; Institute of Environmental Sciences, Jagiellonian University, Kraków, Poland

**Keywords:** Copulation, Courtship, *Drosophila melanogaster*, Ejaculate, Experimental evolution, Mating, Sex peptide, Sexual selection, Sperm competition

## Abstract

**Background:**

The number of partners that individuals mate with over their lifetime is a defining feature of mating systems, and variation in mate number is thought to be a major driver of sexual evolution. Although previous research has investigated the evolutionary consequences of reductions in the number of mates, we know little about the costs and benefits of increased numbers of mates. Here, we use a genetic manipulation of mating frequency in *Drosophila melanogaster* to create a novel, highly promiscuous mating system. We generated *D. melanogaster* populations in which flies were deficient for the sex peptide receptor (*SPR*) gene – resulting in *SPR-* females that mated more frequently – and genetically-matched control populations, and allowed them to evolve for 55 generations. At several time-points during this experimental evolution, we assayed behavioural, morphological and transcriptional reproductive phenotypes expected to evolve in response to increased population mating frequencies.

**Results:**

We found that males from the high mating frequency *SPR*- populations evolved decreased ability to inhibit the receptivity of their mates and decreased copulation duration, in line with predictions of decreased per-mating investment with increased sperm competition. Unexpectedly, *SPR-* population males also evolved weakly increased sex peptide (*SP*) gene expression. Males from *SPR-* populations initially (i.e., before experimental evolution) exhibited more frequent courtship and faster time until mating relative to controls, but over evolutionary time these differences diminished or reversed.

**Conclusions:**

In response to experimentally increased mating frequency, *SPR-* males evolved behavioural responses consistent with decreased male post-copulatory investment at each mating and decreased overall pre-copulatory performance. The trend towards increased SP gene expression might plausibly relate to functional differences in the two domains of the SP protein. Our study highlights the utility of genetic manipulations of animal social and sexual environments coupled with experimental evolution.

**Electronic supplementary material:**

The online version of this article (doi:10.1186/s12862-016-0699-8) contains supplementary material, which is available to authorized users.

## Background

Mating systems in nature range from strict monogamy across a lifespan to mating with many mating partners [1]. Striking natural variation in the number of mates is often observed between closely related species and even within species (e.g., [2]). This variation has important implications for a wide array of evolutionary and ecological processes, including sexual selection, sexual conflict, social organization and evolution, life history evolution, sexually-transmitted pathogen dynamics, speciation, sperm competition and cryptic female choice [3–8]. As a result, there is great potential for variation in the number of mates to drive phenotypic evolution in both sexes. Because the number of mates and mating frequency are the outcome of interacting male and female mating strategies, understanding how mating systems evolve requires characterizing optimal mating strategies in both sexes and how they co-evolve between the sexes.

A powerful tool for investigating the evolutionary consequences of variation in mate numbers is experimental evolution. Several studies have adopted this approach by evolving populations at natural or restricted numbers of partners. These studies have revealed that variation in mating frequency can drive the evolution of a wide range of phenotypes, including mating behaviour [9–12], sperm competitiveness [9, 13], genital and gonad size [14–16], cuticular hydrocarbons [17], mate harming and resistance to harm [18, 19], cognitive function [20], and sex-specific transcription [21].

However, we know little about the evolutionary consequences of increased mating frequency. This is because restricting mating is experimentally tractable, but increasing mating frequency is challenging because high mating frequencies are constrained by limited sexual receptivity in one or both sexes. To overcome this limitation, several studies have experimentally manipulated ecological settings to create conditions favouring higher mating frequencies. For example, mating frequency often increases when the sex with the highest sexual receptivity (typically males) is rarer in a population (e.g., female biased sex ratios [10, 22–27]). These studies demonstrate that when the ecological setting favours increased mating, evolutionary change in mating and life history traits can occur, including increased investment in male reproductive organs [26], increased courtship frequency [28], decreased male ejaculate depletion [26, 29], increased male stimulation of female oviposition after mating [24], increased suppression of female receptivity [30], and decreased male but increased female lifespan [22, 28]. Yet, the evolutionary consequences of increases in mating frequency that are driven by changes in mating behaviour, independent of environmental variation, remain unknown.

Here, we use a genetic manipulation of the fruit fly *Drosophila melanogaster* to experimentally increase mating frequencies in experimental populations. We allowed the populations to evolve under these hyper-promiscuous mating conditions to explore the evolutionary consequences for both sexes. To manipulate mating frequencies, we used flies deficient in the sex peptide receptor (*SPR*) gene [31], which encodes a female receptor for the male seminal protein, sex peptide (*SP* or *Acp70A*) [32, 33]. Sex peptide elicits several post-mating responses in females via the SPR, including inhibiting female sexual receptivity [31]. Thus, females that lack the *SPR* (hereafter *SPR-*) have a greatly reduced refractory period after mating (i.e., they become sexually receptive more quickly) [31]. When *SPR* is removed from populations, males are less able to prevent females from re-mating, creating a novel, highly promiscuous mating system. The increase in mating frequency in *SPR-* populations is within the range of naturally occurring mating frequencies found in closely related, more promiscuous drosophilid species.

We allowed *SPR-* and control populations to evolve for 55 generations. We first confirmed that mating frequencies were elevated in *SPR-* populations and tracked changes in mating frequency over evolutionary time. We then assayed behavioural, morphological and transcriptional phenotypes expected to evolve in response to increased population mating frequencies, at several time points during experimental evolution. Theory suggests that, under increased promiscuity, selection on males should shift from pre-copulatory (e.g., on courtship and mating success) to post-copulatory (e.g., on sperm competition and its avoidance), and that males should decrease per-mating ejaculate investment because the returns from each mating are likely to be lower [34–39]. We therefore tested for experimentally evolved differences in both pre- and post-copulatory phenotypes in males, including courtship behaviour, copulation duration, effects on female post-mating fecundity and re-mating behaviour, testes and accessory gland size, and success in sperm competition. Because males in *SPR*- populations cannot influence female re-mating through SP, male investment in SP should decrease, as should male investment in the related seminal protein *Ductus ejaculatorius peptide 99B* (*Dup99B*), which also binds to SPR [31] and suppresses female post-mating receptivity (although to a lesser degree than SP [32]). To this end, we investigated the expression of both *SP* and *Dup99B* genes using RT-qPCR. We also assayed female mating strategies – including female latency to mating, post-mating fecundity and re-mating behaviour – to determine how the reproductive behaviour of the sexes co-evolves under the novel mating regime.

## Results

We experimentally evolved 4 replicate *SPR-* populations (in which no individuals expressed *SPR*) and 4 genetically matched control populations, from a Dahomey stock into which the *w*^1118^ allele for white eye colour had been backcrossed to facilitate tracking the *SPR* deficiency. We introduced the *SPR* deficiency to the stock population by backcrossing in the deletion *Df(1)Exel6234* ([31]; see also [40]), which carries a *white* + transgene that partially rescues the *w*^1118^ mutation. Thus, the 4 *SPR-* populations bore the *SPR* deficiency and the *w* + transgene and had red eyes, whereas the 4 control populations bore wild-type *SPR* and had white eyes. White-eyed flies have reduced vision relative to wild-type (red) eyes [41]; therefore, the red-eyed *SPR-* flies likely had partially improved visual performance compared with controls. For each replicate population, each generation began with 100 adult males and 100 adult females, which were permitted to interact in chambers for 9 days before progeny were collected for the next generation. Initial tests of male courtship behaviour revealed that *SPR-* and white-eyed control males had comparable courtship rates, whereas wild-type males courted females significantly more (>50 % more frequently) than both *SPR-* and white-eyed males (see Additional file 1: Supporting results). Due to this substantial difference in courtship behaviour we therefore did not include wild-type populations in the subsequent experimental evolution.

For most phenotypes, we first used unselected *SPR-* and control flies to establish baseline differences caused by the *SPR* deficiency itself or by the difference in eye colour between control and *SPR-* flies. Thus, ‘unselected *SPR*-’ and ‘unselected control’ refer to flies that have not undergone experimental evolution, and for which any differences are a result of the genetic manipulation rather than evolution under varying levels of promiscuity. We then tested for evolved differences between the replicate *SPR-* and control populations.

### Behavioural evolution

For most behavioural phenotypes, we first tested for effects of the *SPR* deficiency itself in both sexes. We then tested for evolved differences in pairings between (1) experimentally-evolved males and wild-type females, (2) experimentally-evolved females and wild-type males, and (3) experimentally-evolved males and females within treatments (Table 1).

**Table 1:** Summary of tests for phenotypic differences related to experimental evolution under high promiscuity

Phenotype	Male type	Female type	Generation tested in evolved flies
Behavioural phenotypes			
Mating frequency in population cages	*SPR-*, C	*SPR-*, C	1–17, 28
Courtship frequency	*SPR-*, C	WT	16, 26
Latency to mating	*SPR-*, C	WT	16, 36
	WT	*SPR-*, C	26
	*SPR-*, C	*SPR-*, C	26
Copulation duration	*SPR-*, C	WT	16, 36
	WT	*SPR-*, C	26
	*SPR-*, C	*SPR-*, C	26
Female post-mating fecundity; for males, ability to stimulate fecundity	*SPR-*, C	WT	16
	WT	*SPR-*, C	26
Female latency to re-mating; for males, ability to inhibit re-mating	*SPR-*, C	WT	16, 36
	WT	*SPR-*, C	26
	*SPR-*, C	*SPR-*, C	26
Sperm precedence as first male to mate with a female (P1)	*SPR-*, C	WT	36
Sperm precedence as second male to mate with a female (P2)	*SPR-*, C	WT	36
Morphological evolution			
Male mass	*SPR-*, C	–	16
Testes area	*SPR-*, C	–	16
Accessory gland area	*SPR-*, C	–	16
Gene expression			
*SP* gene expression	*SPR-*, C	–	55
*Dup99B* gene expression	*SPR-*, C	–	55

Phenotypes tested for differences between sex peptide receptor deficient (*SPR-*) flies and controls that expressed *SPR* (C). We tested experimental males with wild-type females, experimental females with wild-type males, or within-treatment tests of *SPR-* or control males and females paired within replicate line

#### Mating frequency

**Fig. 1:**
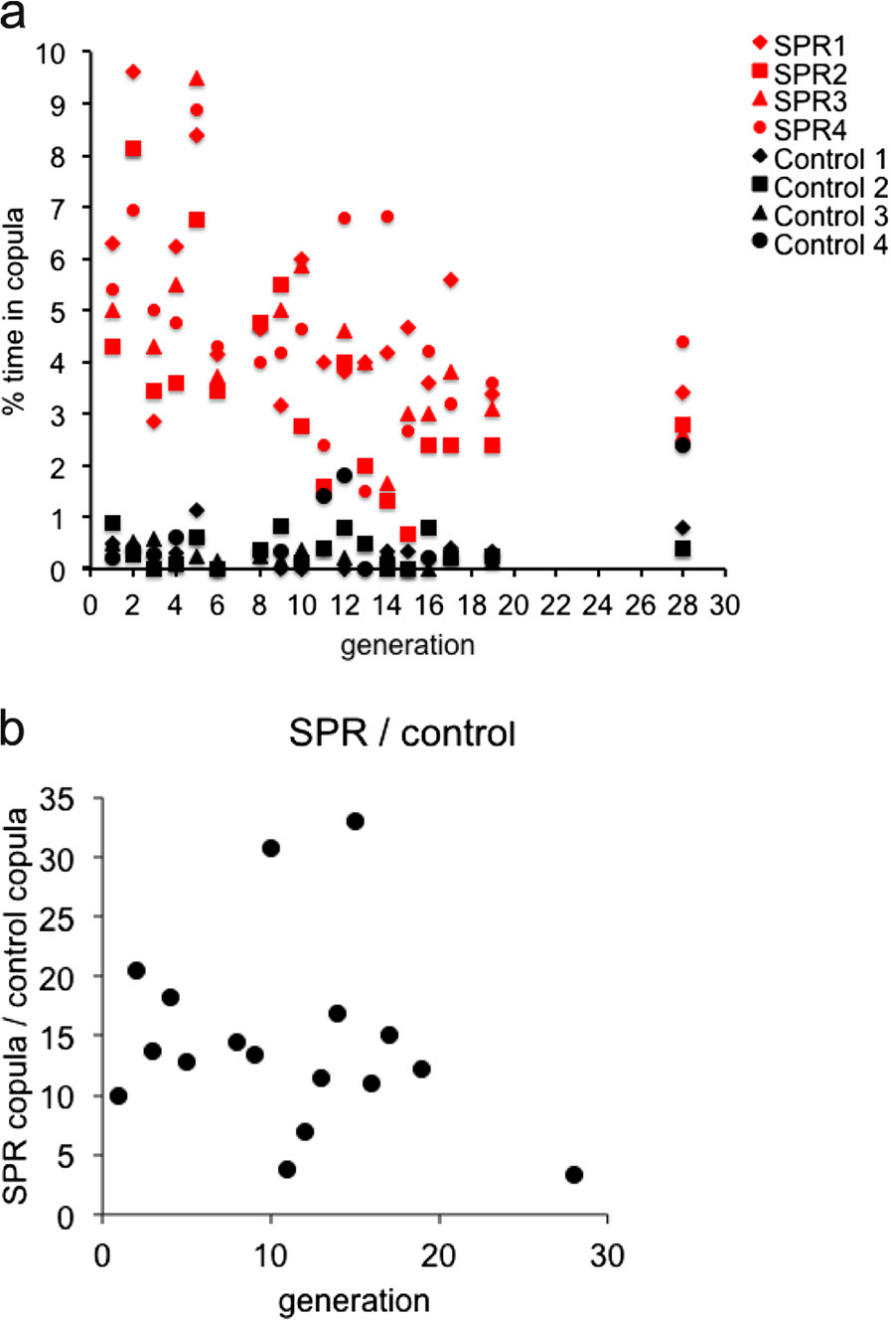
Mating frequency over the course of experimental evolution. (**a**) The percentage of time in which flies were observed mating in replicate populations (1–4) in which flies did (Control) or did not express sex peptide receptor (*SPR-*). (**b**) The proportion of time in copula for *SPR-* populations relative to control populations

As expected, we observed mating ~15-fold more frequently in populations of *SPR*- males and *SPR-* females, compared with control populations (Fig. 1a). This difference was maintained during experimental evolution despite overall declining mating frequency (Fig. 1b).

#### Courtship frequency

Unselected *SPR-* males (i.e., males lacking *SPR*, but not experimentally evolved) courted wild-type females more frequently than did unselected control males (Table 2), likely due to improved vision in *SPR-* populations relative to controls. At generation 16, experimentally-evolved *SPR-* males similarly courted females more frequently than control males, although the difference was not statistically significant (*P* = 0.06). However, at generation 26 we detected no difference nor any trend for experimentally-evolved *SPR-* males to court more frequently (Table 2), suggesting an evolved loss of courtship activity in the hyper-promiscuous *SPR-* lines relative to control lines.

**Table 2:** Male courtship frequency

Test	Generation	*SPR*- mean ± S.E.	Control mean ± S.E.	F	df	P
Effects of *SPR*- deficiency^a^		17.2 ± 0.8	14.7 ± 0.8	4.8	1,77	0.03
Experimental evolution	16	12.2 ± 0.8	9.5 ± 0.8	5.1	1,6	0.06
Experimental evolution	26	18.3 ± 1.6	19.5 ± 1.6	0.3	1,6	0.59

^a^Courtship frequency varied between blocks (block 1: 12.5 ± 0.8 S.E.; block 2: 19.5 ± 0.8 S.E.; F_1,77_ = 27.7, P < 0.0001)

Effects of the *SPR*- deficiency itself in unselected flies, or experimental evolution in *SPR*- and control populations, on the number of courtship events observed during male courtship of wild-type females

#### Latency until mating

Unselected *SPR-* males were faster to mate with wild-type females, at both a male’s first mating and fifth consecutive mating (Table 3), as expected from the relatively better vision of *SPR-* males compared with controls. Although experimentally-evolved *SPR-* males were also faster to achieve first matings at generation 16, we detected no difference at generation 36 nor any trend for *SPR-* males to mate faster. At generation 36, the pattern had reversed, with experimentally-evolved control males being faster than *SPR-* males to achieve a fifth mating (Table 3). This suggests that *SPR-* males lost their mating speed advantage (relative to control males) over evolutionary time.

**Table 3:** Latency until mating

Test	Male type^a^	Female type^a^	Generation	Mating number	Risk ratio, *SPR-*: control (95 % CI)	χ	*P*
(a) Effects of *SPR*- deficiency in males	*SPR-* or C	WT	N/A	1^st^	2.1 (1.0, 4.3)	4.2	0.04
			N/A	1^st^	1.6 (1.0, 2.7)	3.7	0.055
			N/A	5^th^	2.4 (1.4, 4.2)	9.7	0.002
Experimental evolution of males^b^	*SPR-* or C	WT	16	1^st^	2.7 (2.0, 3.6)	43.3	<0.0001
			36	1^st^	1.0 (0.8, 1.2)	0.0	0.82
			36	5^th^	0.8 (0.6, 1.0)	4.2	0.04
(b) Effects of *SPR*- deficiency in females	WT	*SPR-* or C	N/A	1^st^	0.8 (0.5, 1.3)	0.9	0.35
Experimental evolution of females	WT	*SPR-* or C	26	1^st^	0.7 (0.5, 0.9)	5.4	0.02
(c) Effects of *SPR*- deficiency in within-treatment pairs	*SPR-* or C	*SPR-* or C	N/A	1^st^	7.4 (3.5, 16.3)	27.6	<0.0001
Experimental evolution in within-treatment pairs	*SPR-* or C	*SPR-* or C	26	1^st^	3.4 (2.4, 4.8)	47.6	<0.0001

^a^Males and females were *SPR*- or genetically matched controls (C), or wild-type (WT)

^b^Full model details for generation 36 are given in Additional file 1 (Table S1)

Effects of the *SPR*- deficiency itself in unselected flies, or experimental evolution in *SPR*- and control populations, on latency until a first mating by (a) experimental males (mating for the first or fifth time) paired with wild-type females, (b) experimental females paired with wild-type males, or (c) experimental males and females paired within treatment and replicate population. Risk ratios > 1 indicate that *SPR-* males were faster to mate; values < 1 indicate control males were faster

We found no evidence for evolutionary change in latency to mating in either *SPR-* females or in within-treatment pairings. *SPR-* females were faster to mate with wild-type males than control females in both unselected (albeit not significantly so) and experimentally-evolved flies, with a similar magnitude of difference (Table 3). Likewise, *SPR-* males and females paired together were faster to mate than controls in both unselected and experimentally evolved flies (Table 3).

#### Copulation duration

**Fig. 2:**
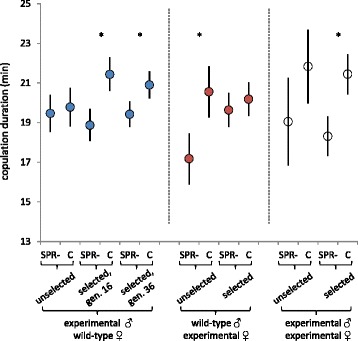
Copulation duration in unselected and experimentally evolved *SPR-* and control flies. Copulation duration measured in flies that either did (control, C) or did not express sex peptide receptor (*SPR-*), for unselected flies (in which any differences between C and *SPR-* flies are caused by the *SPR* deficiency itself) and experimentally-evolved selected flies. Dotted lines demarcate different comparisons where we tested for experimentally evolved differences in males by pairing *SPR-* or C males with wild-type females (at generation 16 or 36), in females by pairing *SPR-* or C females with wild-type males (at generation 26), or in within-treatment pairings of *SPR-* (or C) males with *SPR-* (or C) females (measured at generation 26). Asterisks indicate significant differences between *SPR-* and C flies. Least squares means are presented with 95 % confidence intervals

The *SPR* deficiency itself did not alter male copulation duration in unselected flies, but experimentally-evolved *SPR-* males had shorter copulations than controls (Fig. 2; Table 4). In contrast, the *SPR* deficiency itself caused a shorter female copulation duration, but experimentally-evolved *SPR-* and control females did not differ (Fig. 2; Table 4). In within-treatment pairings, we found only weak evidence that the *SPR* deficiency itself affected copulation duration (Fig. 2; Table 4). However, experimentally-evolved *SPR-* males and females had significantly shorter matings (Fig. 2, Table 4). The above results are for matings between virgin flies; we found no differences in copulation duration in second matings (Additional file 1: Table S2).

**Table 4:** Copulation duration

Test	Male type^a^	Female type^a^	Generation	F	df	*P*
(a) Effects of *SPR*- deficiency in males^b^	*SPR-* or C	WT	N/A	0.2	1,95	0.64
Experimental evolution of males	*SPR-* or C	WT	16	14.6	1,6.2	0.0083
Experimental evolution of males	*SPR-* or C	WT	36	6.6	1,6.8	0.038
(b) Effects of *SPR*- deficiency in females, first mating	WT	*SPR-* or C	N/A	13.5	1,78	0.0004
Experimental evolution of females	WT	*SPR-* or C	26	0.9	1,6.0	0.37
(c) Effects of *SPR*- deficiency in within-treatment pairs	*SPR-* or C	*SPR-* or C	N/A	3.7	1,49	0.059
Experimental evolution in within-treatment pairs	*SPR-* or C	*SPR-* or C	26	67.8	1,6.5	0.0001

^a^Males and females were *SPR*- or genetically matched controls (C), or wild-type (WT)

^b^Copulation duration did not vary between blocks (F_1,95_ = 0.0, P = 0.91)

Effects of the *SPR*- deficiency itself in unselected flies, or experimental evolution in *SPR*- and control populations, on copulation duration in virgin flies for (a) experimental males paired with wild-type females, (b) experimental females paired with wild-type males, or (c) experimental males and females paired within treatment and replicate population

#### Post-mating fecundity

Neither unselected nor experimentally-evolved *SPR-* and control males differed in their ability to stimulate female oviposition (Additional file 1: Table S3). When expressed in females, the *SPR* deficiency itself reduced fecundity in unselected flies, as expected given SP’s role in fecundity stimulation [42] (Additional file 1: Table S3). Although we hypothesized that experimentally evolved *SPR-* females would evolve relatively increased post-mating fecundity, through selection to decrease reliance on SP, the difference in egg-laying at generation 26 was similar to that caused by the *SPR* deficiency in unselected flies (Additional file 1: Table S3).

The *SPR-* deficiency did not alter male fertility (proportion of eggs developing into adults: median and range, *SPR-*, 0.80 [0.29, 0.98]; control, 0.80 [0.00, 0.92]; Wilcoxon χ^2^_1_ = 0.2, *P* = 0.66), suggesting that fertility differences did not confound the sperm competition experiments (results below).

#### Re-mating behaviour

As expected given that SPR is not known to impact male ejaculate parameters, the *SPR* deficiency did not affect male ability to inhibit re-mating by their wild-type mates, whether measured as latency to re-mating (Table 5) or (in a separate experiment) as the number of females re-mating (generation 0: initial mating with *SPR-* male: 11/20; initial mating with control male: 9/18; χ^2^_1_ = 0.1, *P* = 0.76). Although experimentally-evolved males likewise did not differ in ability to inhibit re-mating at generation 16, *SPR-* males were significantly worse at inhibiting re-mating at generation 36 (Table 5).

**Table 5:** Re-mating behaviour

Test	Male type^a^	Female type^a^	Generation	Risk ratio, *SPR-*:control (95 % CI)	χ	*P*
(a) Effects of *SPR*- deficiency in males^b^	*SPR-* or C	WT	N/A	0.6 (0.3, 1.3)	1.7	0.19
Experimental evolution of males, generation 16	*SPR-* or C	WT	16	1.2 (0.8, 1.9)	0.9	0.35
Experimental evolution of males, generation 36^b^	*SPR-* or C	WT	36	1.4 (1.0, 1.8)	4.2	0.04
(b) Effects of *SPR*- deficiency in females	WT	*SPR-* or C	N/A	1.4 (0.9, 2.3)	2.2	0.14
Experimental evolution of females	WT	*SPR-* or C	26	2.8 (2.0, 4.1)	30.6	<0.0001
(c) Effects of *SPR*- deficiency in within-treatment pairs	*SPR-* or C	*SPR-* or C	N/A	2.6 (1.3, 5.1)	7.7	0.006
Experimental evolution in within-treatment pairs	*SPR-* or C	*SPR-* or C	26	2.3 (1.6, 3.3)	20.7	<0.0001

^a^Males and females were *SPR*- or genetically matched controls (C), or wild-type (WT)

^b^Full model details are given in Additional file 1: Table S4

Effects of the *SPR*- deficiency itself in unselected flies, or experimental evolution in *SPR*- and control populations, on (a) experimental male ability to inhibit re-mating by wild-type females, or (b, c) experimental female propensity to re-mate with wild-type males following a mating with a (b) wild-type male or (c) experimental male of the same treatment group

Surprisingly – given that SP inhibits female re-mating – when the *SPR-* deficiency was expressed in unselected females, we did not detect significant changes in latency until re-mating (Table 5); nonetheless, the confidence interval for the difference between groups is 0.9 – 2.3 (where 1 denotes identical risk ratios), indicating no strong support for rejecting the null hypothesis of no difference. However, experimentally-evolved *SPR-* females re-mated more quickly after mating with wild-type males (Table 5). In within-treatment pairings, we found the expected faster re-mating following matings between *SPR-* males and *SPR-* females, compared with control males and control females, with a similar magnitude of difference for both unselected and experimentally-evolved flies (Table 5).

### Morphological evolution

Evolving in populations that lacked *SPR* had no significant effect on testes or accessory gland size – neither on absolute organ size nor on body mass-corrected size – or on body mass, nor did the *SPR* deficiency itself influence these traits (Additional file 1: Table S5). We tested whether the *SPR* deficiency influenced female mass as a test for pleiotropic effects of the deficiency, but found no difference (Additional file 1: Table S5).

### Ejaculate evolution

#### Sperm precedence as first male to mate

Neither the *SPR* deficiency itself nor evolution in *SPR-* populations affected either male success in sperm competition as the first male to mate (P1) or male ability to maintain P1 over multiple matings (Table 6).

**Table 6:** Sperm precedence

Phenotype	Test	Generation	Mating number	*SPR*- proportion sired ± S.E.	Control mean proportion sired ± S.E.	t-value	df	*P*
P1^a^	Effects of *SPR*- in unselected males	N/A	1^st^	0.06 ± 0.04	0.15 ± 0.03	3.3	1,36	0.08
			5^th^	0.04 ± 0.01	0.05 ± 0.01	0.1	1,36	0.82
	Effects of *SPR-* in experimentallly-evolved backcrossed controls	N/A	1^st^	0.17 ± 0.01	0.12 ± 0.01	−1.0	1,284	0.31
			5^th^	0.07 ± 0.01	0.11 ± 0.01	−0.7	1,224	0.47
	Experimental evolution of males	36	1^st^	0.14 ± 0.02	0.18 ± 0.02	−1.4	1,6	0.20
			5^th^	0.06 ± 0.01	0.08 ± 0.01	−1.5	1,6	0.19
P2^b^	Effects of *SPR*- in unselected males^b^	N/A	1^st^	0.88 ± 0.03	0.89 ± 0.04	0.2	1,35	0.60
	Effects of *SPR-* in experimentally-evolved backcrossed controls	N/A	1^st^	0.78 ± 0.12	0.91 ± 0.06	2.5	1,221	0.01
	Experimental evolution of males	36	1^st^	0.85 ± 0.02	0.81 ± 0.02	2.2	1,6	0.07

^a^Full model results in Additional file 1: Table S6

^b^Full model results in Additional file 1: Table S7

Effects of the *SPR*- deficiency itself, or experimental evolution in *SPR*- and control populations, on the proportion of offspring sired by a focal male as the first or second male to mate with a wild-type female (P1 or P2, respectively). P1 tests include a male’s first and fifth mating to test for differences in male ejaculate depletion across successive matings. Effects of the *SPR* deficiency itself were evaluated in unselected flies or in experimentally evolved backcrossed or outcrossed controls (see Methods)

#### Sperm precedence as second male to mate

We found mixed evidence that the *SPR* deficiency itself affected male success in sperm competition as the second male to mate (P2), but experimentally evolved *SPR-* and control males showed little difference (Table 6).

#### Sex peptide and Dup99B gene expression evolution

**Fig. 3:**
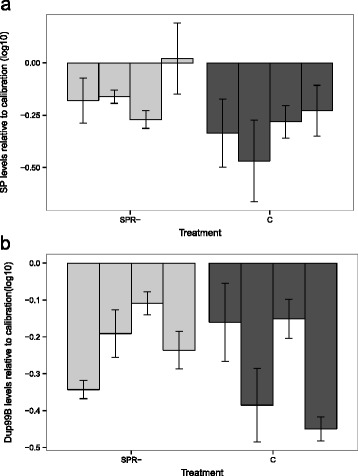
Experimental evolution of *sex peptide* (*SP*) and *Dup99B* gene expression. Mean levels of gene expression of (**a**) *SP* and (**b**) *Dup99B* in four experimentally evolved replicate populations each of the sex peptide receptor-deficient (*SPR-*) treatment (in which neither sex expressed *SPR*) and the control treatment. Gene expression was measured at generation 55. Error bars represent ± 2 S.E

Surprisingly, we found a trend towards higher *SP* expression in experimentally evolved *SPR-* males, compared with controls (F_1,6_ = 5.1, P = 0.06; Fig. 3a). In contrast, there was no significant difference in *Dup99B* expression (F_1,6_ = 0.5, P = 0.49; Fig. 3b). Characteristics of the RT-qPCR calibration curves are given in Additional file 1 (Table S8).

## Discussion

Our study investigated the evolutionary consequences of increased mating frequencies above naturally occurring levels. Our main findings are that, after evolution at high mating frequency in *SPR-* populations, males from these populations evolved shorter copulation duration and decreased ability to inhibit female re-mating. These changes are consistent with decreased male post-copulatory investment at each mating. Surprisingly, males from *SPR-* populations also tended to show an evolved increase in *SP* gene expression, and we discuss hypotheses that might explain this pattern below. Furthermore, although *SPR-* males initially courted females more frequently and mated faster than controls, we found that after experimental evolution these differences were no longer detectable (for courtship frequency and time until first mating) or had reversed (for time until fifth mating), results consistent with decreased pre-copulatory investment.

### Reduced ejaculate investment

Sperm competition theory predicts that males should decrease their investment in ejaculate at each mating as the intensity of sperm competition (i.e., the number of competing ejaculates from different males) increases because the returns from investing in a given mating decrease [34–38]. We confirmed that mating frequency was increased in *SPR-* populations, such that sperm competition would have been intensified if increased mating frequency resulted in greater temporal overlap of ejaculates from multiple males (e.g., [43]) . This is an especially likely scenario here because SPR regulates female sperm storage, such that *SPR-* females retain more sperm in the days after mating [44].

Two lines of evidence support the prediction that per-mating ejaculate investment decreased in males from *SPR-* populations. First, *SPR-* males evolved decreased copulation duration compared with control males. Copulation duration in matings with virgin females is positively correlated with ejaculate transfer in *Drosophila* spp. ([45, 46]; but see [47]), suggesting that *SPR-* males might transfer less ejaculate per mating. Second, *SPR-* males were less able to inhibit female re-mating, consistent with the hypothesis that they transfer fewer receptivity-inhibiting seminal proteins. Below, we discuss hypotheses to reconcile this result with our finding of increased *SP* gene expression for these males. Although these results are consistent with decreased per-mating ejaculate investment, we did not find differences in either male stimulation of female fecundity or success in sperm competition. Low statistical power is a potential concern, but our sample sizes were sufficient to allow detection of some effects of the *SPR* deficiency itself on these traits. Taken together, these changes in ejaculate-related traits are consistent with decreased investment in some ejaculate components more than others and a shift in ejaculate composition [48].

### *Expression of* sex peptide

If expressing *SP* is costly, then males co-evolving with *SPR*- females should evolve lower *SP* gene expression because SP is functionless with these females. There are several potential explanations for our finding that *SP* expression was weakly increased, in addition to the possibility that the marginally non-significant result (*P* = 0.06) arose by chance. First, expressing *SP* may not be costly for males; however, this alone does not explain increased *SP* expression in *SPR-* males. Second, *SP* expression measured in males might not reflect SP protein transfer to females at mating. Indeed, a recent study found no correlation between the two [49]. *SP* expression in males could instead reflect male SP stores, with larger stores evolving to increase male capacity to mate many times in *SPR-* populations. However, we detected no change in accessory gland size, nor evidence that *SPR-* males could better sustain ejaculate transfer over multiple matings, as *SPR-* and control males had similar success in sperm competition in first and fifth matings.

Third, if *SP* expression is in fact positively correlated with SP protein transfer, then increased *SP* expression in *SPR-* males appears inconsistent with our finding of decreased ability of *SPR-* males to inhibit female re-mating. However, if functionality of the SP protein degraded through genetic drift, a likely scenario given that SP is effectively neutral in *SPR*- lines, this inconsistency is reconciled. Under this scenario, there are two potential explanations for increased *SP* gene expression. First, *SP* expression might be genetically correlated with other ejaculate components under positive selection (e.g., to compensate for SP in *SPR-* populations). A second mechanism relates to SP protein structure, which consists of a carboxy-terminal end that binds to SPR and mediates female post-mating oviposition and re-mating responses, and an amino-terminal end that does not bind to SPR but to unknown female receptors [31]. Positive selection on the amino-terminal end might occur in *SPR-* populations to compensate for the lack of oviposition stimulation from the carboxy-terminal end, because the amino-terminal end induces juvenile hormone synthesis in adult females and stimulates oogenesis ([50, 51]; see also [52]). We did not find that *SPR-* males increased the post-mating fecundity of wild-type females, although the wild-type female fecundity response might be dominated by carboxy-terminal end effects. This hypothesis is consistent with our finding of no increase in *Dup99B* expression, as *Dup99B* is homologous with *SP* only at the carboxy-terminal end. Finally, *SP* might have other as yet unknown functions that are selected positively under high promiscuity.

### Evidence for decreased pre-copulatory performance

We found two effects on pre-copulatory performance that were most likely caused by the improved vision of red-eyed *SPR-* males relative to white-eyed controls: unselected *SPR-* males courted females more frequently and were faster to mate. Both effects remained detectable at generation 16, but by generation 36, both were either not statistically significant or significant and reversed (with control males faster to achieve a fifth mating). Although type II error is a possibility such that we simply failed to detect the same differences, the trends were no longer in the previous direction: at generation 36, mean courtship frequency was higher in control males and the risk ratio for latency to mating was 1.0. The results are consistent with decreased pre-copulatory investment in *SPR-* males, which might result from weaker selection to overcome re-mating resistance with *SPR-* females. Declining courtship frequency might be accompanied by decreased courtship intensity or quality of courtship signals. Furthermore, if the increased mating frequency in *SPR-* populations caused sexual selection to shift from pre-copulatory to post-copulatory, we would expect increased displays of post-mating guarding behaviour (recently reported in *D. melanogaster* [53]) in *SPR-* males. We are currently investigating these hypotheses.

## Conclusions

Our study shows that *SPR-* males evolving at high promiscuity evolve shorter copulations and decreased inhibition of female re-mating, consistent with decreased post-copulatory investment per mating, and less frequent courtship and longer latency until mating, consistent with decreased pre-copulatory investment. Curiously, males that evolved with *SPR-* females also showed a tendency for increased *SP* expression. Previous experimental evolution studies have demonstrated that reducing a population’s mating frequency by imposing monogamy can result in evolutionary change in reproductive traits (e.g., [10, 23, 54]). Other studies have increased or decreased mating frequency by altering the sex ratio (e.g., [24–26, 28]), but changes in sex ratio tend to alter mating frequency and mate competition in opposite directions (e.g., with fewer matings but more intense competition at male-biased sex ratios). The effects of increased promiscuity *per se*, as observed in our study, seem to be different from simultaneously changing mating frequency and mate competition. Our study highlights the utility of genetic manipulations of the socio-sexual environment, and the sensitivity of experimental evolution studies of mating system to the method by which mating frequency is manipulated.

## Methods

### Fly stocks and experimental populations

All flies shared an outbred, lab-adapted Dahomey genetic background. To set up the control and experimental populations, we first generated a white-eyed Dahomey background population by backcrossing a loss of function allele (*w*^1118^) for the X-linked white gene into the Dahomey background, yielding white eyes [55]. We then derived the *SPR-* populations by backcrossing the deletion mutation *Df(1)Exel6234* ([31]; see also [40]), which covers the entire X-linked *SPR* gene and four linked loci, into the white-eyed Dahomey background population for 5 generations (Additional file 2: Figure S1). The mutation carries a white^+^ transgene, which provides a partial rescue of the *w*^1118^ mutation. Thus, in a *w*^1118^ background, male hemizygote and female homozygote carriers of *Df(1)Exel6234* possess red eyes, whereas heterozygote females possess orange eyes, which facilitates tracking the *SPR* deficiency.

We used these populations to establish 4 replicate *SPR*- populations that did not express *SPR* and 4 genetically-matched control populations, for a total of 8 populations [40] (Additional file 2: Figure S1). Replicate populations were maintained for 36 generations as follows. Each generation began with 100 adult males and 100 adult females permitted to interact in 4.5 L chambers for 9 days. Each chamber contained three bottles of food media (50 mL) with granules of live yeast. Bottles were replaced on days 4 and 7. After 9 days, eggs were collected for propagation of the next generation and transferred at standardized density to bottles. Adults were collected 13 days later. From generations 36–45, populations were maintained by transferring adults to new food every two weeks at an uncontrolled sex ratio and density. From generations 0–45, flies were maintained and experiments conducted on sugar-yeast-molasses medium in plastic bottles or vials at 25 °C on a 12:12 h light:dark cycle.

At generation 45, each replicate population was divided among 10 vials with Jazz-mix food medium (Fisher Scientific), with 3 males and 3 females per vial. Virgin progeny were collected and housed in sex-specific vials. For each population, progeny from all 10 vials were pooled and 30 males and 30 females were used to start the next generation. Three virgin males and 3 virgin females were housed per vial for a two-day oviposition period, after which they were removed and offspring were allowed to develop for 10–11 days.

We conducted two further rounds of backcrossing *Df(1)Exel6234* into the white-eyed Dahomey background population to generate unselected *SPR-* and control flies that had not undergone experimental evolution (Additional file 2: Figure S1), in order to further test for differences caused by the deficiency itself at generations 26 and 36 (in addition to initial tests at generation 0).

To conduct the sperm competition experiments (at generation 36), we established additional populations to aid in distinguishing evolved responses from effects of the *Df(1)Exel6234* deficiency itself. These populations consisted of (1) four ‘*SPR+* evolved control’ populations, in which the genetic background was that of populations that evolved with females expressing *SPR*, but through backcrossing they carried *Df(1)Exel6234* and had red eyes; or, conversely, (2) four ‘*SPR-* evolved control’ populations, in which the genetic background was that of populations that evolved without *SPR* expression, but through backcrossing they carried wild-type *SPR* and had white eyes (Additional file 2: Figures S2 and S3). We refer to these ‘*SPR-* evolved control’ and ‘*SPR+* evolved control’ populations together as ‘experimentally-evolved backcrossed controls’. To generate the four *SPR-* evolved control populations, beginning at generation 26, we backcrossed *Df(1)Exel6234* into the four control populations (i.e., the four control populations as described above, having undergone 26 generations of experimental evolution under controlled conditions). We did this by crossing virgin females from each control population with males from an unselected *SPR-* population. Heterozygote orange-eyed virgin daughters were backcrossed with males from each respective (parental) control population. After 6 generations of backcrossing, orange-eyed daughters were crossed with red eyed males from the same replicate for 4 generations to generate red eyed progeny (Additional file 2: Figure S2). We conducted analogous backcrossing to generate the four *SPR+* evolved control populations (Additional file 2: Figure S3).

In the sperm competition experiments, we used competitor males that were homozygous for the recessive sparkling (*spa*) mutation, which causes a rough eye phenotype in homozygotes, backcrossed into a Dahomey background.

### Tests of experimentally evolving phenotypes

We conducted five sets of experiments (Table 1): (1) initial tests using unselected flies that had not undergone experimental evolution (i.e., immediately after backcrossing, at generations 0, 26 and 36 as described above), to establish baseline differences and test for effects of the *Df(1)Exel6234* deficiency itself (a necessary step, as differences between control and *SPR-* flies might occur due to the difference in eye colour used a phenotypic marker for the deletion, pleiotropic effects of *SPR* in either sex, or effects of the four genes linked to *SPR-* in the *Df(1)Exel6234* deficiency; 31); (2) at generation 16, tests of evolved male phenotypes, by pairing experimentally evolved males with standard wild-type females which are described below; (3) at generation 26, tests of evolved female phenotypes, by pairing experimentally evolved females with standard wild-type males, and tests of co-evolved male and female traits, by pairing male and females of the same treatment; (4) at generation 36, tests of sperm competitiveness; and (5) at generation 55, tests of the evolution of *SP* and *Dup99B* gene expression. Standard wild-type males and females were derived from the Dahomey stock population maintained in our laboratory in population cages housing thousands of flies. All flies used in the behavioural, morphological and sperm competition experiments, including standard wild-type and experimental flies, were reared at standard density by allowing females to oviposit on petri dishes containing agar-grape juice medium, from which first instar larvae were transferred to bottles containing standard food, at a density of 100 larvae/7.5 mL food. Flies were collected as virgins within six hours of eclosion using ice anesthesia, housed in same-sex vials containing food medium and live yeast in groups of 15–20 for males and 10–15 for females, transferred to fresh vials every 2–3 days, and were 2–5 days post-emergence when used in experiments.

### Behavioural evolution

Except where stated, we transferred males to individual vials containing food medium and live yeast approximately 20 h before experiments began, and added females to male vials to begin each experiment. We excluded individuals for which copulation duration was less than five minutes because it is unclear whether these represent real copulations involving ejaculate transfer (e.g., [56, 57]).

#### Mating frequency

In 18 generations between generations 1 and 28 (17 of the first 19 generations, plus generation 28), we conducted spot check observations of male–female interactions for each replicate population (Table 1). Most observations were taken on days 2–5 of the interaction period. Over the 18 generations a total of 141 observations were taken and 2716 matings were observed.

#### Courtship frequency

We tested for differences in courtship frequency caused by either the *SPR* deficiency itself (tested in two blocks) or by experimental evolution (Table 1), by placing 5 Dahomey wild-type virgin females with five virgin males of either *SPR-* or control type in a vial containing food media and live yeast. We assayed multiple replicate vials in each trial, such that vial was the unit of replication (for effects of the *SPR* deficiency: *N* = 17–20 per group in block 1, *N* = 31 per group in block 2; for experimentally evolved differences: *N* = 10 per group). We conducted spot check observations several times daily over four days, in which we recorded the number of courtship events per vial.

#### Latency until mating

We tested for differences in the latency until mating in virgin flies of both sexes, caused by either the *SPR* deficiency itself or experimental evolution (Table 1), by pairing *SPR*- or control males (or females) with virgin wild-type Dahomey females (or males). We also tested for differences in within-population pairings of experimentally evolved males and females, in which *SPR-* males and females were paired together and control males and females were paired together. We also measured latency to mating as part of the sperm competition experiments (see below).

#### Copulation duration

We measured copulation duration for a first mating between virgin flies in the experiments conducted to measure latency until mating described above (Table 1). We also measured copulation duration in a second mating in the experiments involving *SPR-* and control females paired with wild-type males, and for within-treatment pairings (described above), by pairing females with a new male after their initial mating. We also measured copulation duration for virgin and previously-mated females as part of the sperm competition experiments (see below).

#### Post-mating fecundity and fertility

We tested for differences in the ability of male *SPR-* and control males to stimulate egg-laying by wild-type Dahomey females, and for differences in egg-laying by *SPR-* and control females following mating with a wild-type male (Table 1). In both experiments, we counted the number of eggs females laid in individual vials containing food medium in the 24 h following a single mating. At generation 0, we also tested for differences in male ability to fertilize eggs caused by the deficiency alone. We measured fertility as the proportion of eggs developing into adults, by counting adult offspring 12 days after females were removed from vials. We measured egg fertility to ensure that differences in fertility were not a confounding factor in the sperm competition experiments.

#### Latency until re-mating

We tested for differences in latency to re-mating for (1) *SPR-* and control male ability to inhibit re-mating by wild-type Dahomey mates, (2) *SPR-* and control female propensity to re-mate with wild-type Dahomey males, and (3) within-population pairings. We measured these responses as part of the experiments described above (see ‘Latency until mating’).

To measure latency to re-mating in females after matings with *SPR-* or control males, we separated females from males following a single mating. Females were housed for 24 h in individual vials and allowed to oviposit so that we could measure egg production. After 24 h, we transferred females to a new vial containing food medium and a virgin wild-type Dahomey male. We recorded the time of transfer and the time mating began. We followed a similar procedure to measure *SPR-* and control female propensity to re-mate with a wild-type male, following an initial mating with a wild-type male. We also followed a similar procedure in the within-population experiment to measure *SPR-* and control female propensity to re-mate with a wild-type male, following an initial mating with a male of the same replicate population as the female. We also measured latency to re-mating as part of the sperm competition experiments (see below).

### Morphological evolution

We tested for differences in male mass and male testes and accessory gland size that were caused by the deficiency itself (in unselected flies) or the result of experimental evolution (Table 1). We also measured female mass in unselected flies to test for pleiotropic effects of the *SPR* deficiency. To measure male and female mass, we weighed virgin flies to the nearest 10^−3^ mg. To measure testes and accessory gland size, we dissected testes and accessory glands from virgin males in phosphate-buffered saline (PBS), mounted them individually on microscope slides, photographed them along with a one-millimeter measure, and calculated their area from the digital photographs using ImageJ software [58]. We measured both testes and calculated the mean area per testis, and likewise calculated mean area per accessory gland.

### Ejaculate evolution

#### Sperm competitiveness

We tested male sperm competitiveness when males were first or second to mate with a female (i.e., in the disfavoured P1 and favoured P2 roles, respectively) in two separate experiments. In the P1 experiment, we tested male sperm competitiveness when males mated for the first time or fifth consecutive time, to test male responses when ejaculate stores were full or depleted, respectively. We tested unselected *SPR-* and control males that had not undergone experimental evolution to test for effects of the *Df(1)Exel6234* deficiency itself, as well as experimentally evolved *SPR*- and control males, and males from the experimentally-evolved backcrossed control populations described above..

In each experiment, focal males competed against competitor males that were homozygous for the recessive sparkling (*spa*) mutation, which causes a rough eye phenotype in homozygotes. Females in the sperm competition experiments were also homozygous for *spa*, thus allowing us to identify offspring fathered by the experimental or competitor males by eye phenotype [59]. In the P1 experiment, focal males were mated with females on day one of the experiment. Females were then transferred individually to new vials and *spa* competitors were introduced for re-mating on the following day. When females did not re-mate on the second day, the *spa* male was removed and a new *spa* male was introduced on the third day. We repeated the experiment to constitute two blocks. In the P2 experiment, we followed a similar procedure, with *spa* males mated with females on day one of the experiment and focal males introduced for re-mating on the following day. We conducted a single replicate of this experiment. In both experiments, we recorded latency to mating and copulation duration.

After an initial mating, the first and 5th females to mate with a male were transferred individually to separate new vials containing food media and yeast granules and allowed to oviposit for 24 h. We scored adult offspring 12 days later.

#### Sex peptide and Dup99B gene expression

To prepare flies for measurement of gene expression, at generation 55 we standardized larval density within each replicate population by pairing 3–4 day old virgin males and females for 24 h, with one pair per vial in each of 15 vials. Males were then removed and females permitted to oviposit on agar-grape juice plates. Eggs were transferred to vials containing food medium (40 eggs per vial, 5 vials per replicate population). Emerging male offspring were collected as virgins and housed individually.

Four virgin males, each 6 days old, from each replicate population were dissected and their abdomens stored individually in RNA*later* reagent (Sigma-Aldrich) at 4 °C for 24 h and then at −80 °C. Prior to RNA extraction, the abdominal tissue was frozen using liquid nitrogen and homogenized. Total RNA was extracted using a Purelink RNA mini kit (Ambion) using ethanol and 2-mercaptoethanol and an on-column DNase treatment (Purelink DNase I). RNA was eluted with RNA storage solution (Ambion) and stored at −80 ° C. RNA yield (quantified with Qubit 2.0 Fluorometer, Invitrogen) ranged between 3–6 μg/ml.

RT-qPCR assays were set up manually using Brilliant III Ultra-Fast SYBR Green QRT-PCR Master Mix (Stratagene, Agilent Technologies) on an Applied Biosystems 7500 Fast Real-Time PCR system. Amplification reactions were performed in 20 μl total volume with 2 μl of RNA and 0.1 μM of each primer, under the following conditions: incubation at 50 °C for 2 min, 95 °C for 10 min, followed by 40 cycles of 95 °C for 15 s and 60 °C for 1 min.

We used three technical replicates for each of the 4 biological replicate samples per population. *RpL32* was the reference gene. Relative standard curves were generated with serial dilutions of RNA (1, 1/5, 1/25, 1/125, 1/625). Stock RNA for the relative standard curves was extracted from pooled whole males from the four control populations. For the calibrator sample, RNA from abdominal tissues of 15 males from each of the four control populations was pooled and diluted 10 times. Triplicate reactions of the calibrator, no-template control and no-reverse transcriptase control were used on each PCR plate. Primers for *SP*, *Dup99B*, and *RpL32* were designed using NCBI Primer-BLAST (Additional file 1: Table S9) and manufactured by Genomed, Poland. The primers covered exon–intron boundaries (i.e., each primer had sequences from two exons).

Raw data were obtained from the Sequence Detection Systems Software v1.3 as mean values and standard deviations across technical replicates of the target and reference genes. Raw data were normalized using the relative standard curve method. For each biological replicate and for the calibrator sample on each plate, the mean quantity of *SP* or *Dup99B* was normalized to the mean quantity of *RpL32*. The fold difference between treatment sample and calibrator was calculated as the normalized value of the sample divided by the normalized value of the calibrator.

### Statistical analyses

We tested for differences in courtship, copulation duration, post-mating fecundity, testes and accessory gland area, and body mass-corrected testes and accessory gland area that were related to the deficiency itself, in unselected flies, using linear models in which the fixed factor treatment had the levels *SPR-* and control. We tested for differences in these phenotypes in experimentally evolved flies using similar mixed models with the additional random factor of replicate population nested within treatment. We transformed responses where necessary to meet the assumptions of parametric statistics, and used a Wilcoxon test if no transformation was sufficient. Least squares means are presented with standard errors unless otherwise noted. We tested for differences in latency until mating and re-mating using proportional hazards survival models including the factor treatment (for unselected flies) or treatment and replicate population nested in treatment as a random factor (for experimentally evolved flies). Linear and survival models were performed in JMP v. 11.2.0 (SAS Institute). To analyze male success in sperm competition, we used generalized linear models specifying a quasibinomial link function, implemented in R v3.02 [60] using the MASS package [61]. Models for paternity in the P1 experiment included the factors treatment, presence of the *SPR* deficiency (because experiments included the additional experimentally-evolved backcrossed controls described above), the day on which re-mating occurred (i.e., the 2^nd^ or 3^rd^ day of the experiment), copulation durations in the first and second matings, block, and replicate population nested within treatment. Models for paternity in the P2 experiment included the factors treatment, presence of the *SPR* deficiency, copulation durations in the first and second mating, and replicate population nested within treatment. To examine the effect of the experimental evolution treatment on expression levels of *SP* and *Dup99B* in males from *SPR-* and control populations, we used mixed models as above, with the fixed factor treatment and the random factor replicate population nested within treatment. Fold difference values were log-transformed to meet the assumptions of the linear model.

## Abbreviations

P1, Proportion of offspring sired by the first of two males to mate with a female; P2, Proportion of offspring sired by the second of two males to mate with a female; RT-qPCR, Reverse transcription quantitative polymerase chain reaction; SP, sex peptide; SPR, sex peptide receptor

## Additional files

Additional file 1: Supporting results. Courtship behaviour results for unselected *SPR-*, white-eyed control and wild-type control males. **Table S1.** Full statistical results for latency until mating in the sperm competition experiments. **Table S2.** Copulation duration results in second matings. **Table S3.** Results for post-mating fecundity. **Table S4.** Full statistical results for latency until re-mating in the sperm competition experiments. **Table S5.** Results for male and female body mass and both absolute and size-corrected male testes and accessory gland size. **Table S6.** Full statistical results for proportion of offspring sired when a male was first to mate. **Table S7.** Full statistical results for proportion of offspring sired when a male was second to mate. **Table S8.** Details of the RT-qPCR calibration curve. **Table S9.** Details of primer characteristics. (DOCX 67 kb) Additional file 2: Figure S1. The backcrossing procedure used to generate the experimental populations. **Figure S2.** The backcrossing procedure used to generate SPR+ evolved controls for the sperm competition experiments. **Figure S3.** The backcrossing procedure used to generate SPR- evolved controls for the sperm competition experiments. (PPT 368 kb)
