# Supplementary material for: Experimental evolution under hyper-promiscuity in Drosophila melanogaster
Source: BMC Evol Biol. 2016 Jun 16;16:131. doi: 10.1186/s12862-016-0699-8 (PMC4910217; doi:10.1186/s12862-016-0699-8)
Supplement: Additional file 1: — Supporting results. Courtship behaviour results for unselected SPR-, white-eyed control and wild-type control males. Table S1. Full statistical results for latency until mating in the sperm competition experiments. Table S2. Copulation duration results in second matings. Table S3. Results for post-mating fecundity. Table S4. Full statistical results for latency until re-mating in the sperm competition experiments. Table S5. Results for male and female body mass and both absolute and size-corrected male testes and accessory gland size. Table S6. Full statistical results for proportion of offspring sired when a male was first to mate. Table S7. Full statistical results for proportion of offspring sired when a male was second to mate. Table S8. Details of the RT-qPCR calibration curve. Table S9. Details of primer characteristics. (DOCX 67 kb) [file 12862_2016_699_MOESM1_ESM.docx]

**Supplementary information**

**Courtship behavior of unselected *SPR-*, white-eyed control and wild-type control males**

In generation 1 (i.e., ‘unselected flies’, prior to experimental evolution), we compared the courtship behavior of *SPR-*, white-eyed, and Dahomey wild-type (red-eyed) males, in order to determine suitable controls for the experimental evolution populations. We raised flies at standard density and collected virgin adult flies using the standard methods described in the main text. At 3-5 days post-eclosion, we placed in each yeasted vial 5 experimental males with 5 wild-type Dahomey females. We used 20 vials per treatment, and recorded male-female courtship events over 17 observations spread over 4 days. Courtship data was analysed using linear models including male type (i.e., *SPR-*, white-eyed or wild-type) and day as fixed factors, and vial number as a random effect.

We observed the following rates of courtship events per observation per vial (± 95% confidence intervals): wild-type males, 1.14 (1.0, 1.281); *SPR*- males, 0.744 (0.60, 0.884); white-eyed males, 0.716 (0.58, 0.86). Thus, wild-type males had significantly higher courtship rates than either *SPR-* or white-eyed controls. Based on these results, we used white-eyed flies rather than wild-type flies as controls during the experimental evolution.

**Table S1. Latency until mating**

Full model results for latency until mating for (a) experimentally evolved *SPR-* and control males paired with wild-type females for a first mating, (b) experimentally evolved *SPR-* and control males paired with wild-type females for a fifth consecutive mating, (c) unselected *SPR-* and control males paired with wild-type females for a first mating, and (d) unselected *SPR-* and control males paired with wild-type females for a fifth mating

**(a) Experimentally evolved males at generation 36: first mating**

| Factor | d.f. | χ^2^ | P | Risk Ratio | Lower CI | | Upper CI |
| --- | --- | --- | --- | --- | --- | --- | --- |
| Treatment | 1 | 0.051 | 0.822 | 0.9762 | 0.788 | 1.208 | |
| SPR deficiency | 1 | 0.559 | 0.455 | 1.086 | 0.874 | 1.347 | |
| Block | 1 | 40.57 | <0.0001 | – | – | – | |

**(b) Experimentally evolved males at generation 36: fifth mating**

| Factor | d.f. | χ^2^ | P | Risk Ratio | Lower CI | | Upper CI |
| --- | --- | --- | --- | --- | --- | --- | --- |
| Treatment | 1 | 4.239 | **0.040** | 0.753 | 0.575 | 0.986 | |
| SPR deficiency | 1 | 19.9 | **<0.0001** | 1.853 | 1.415 | 2.425 | |
| Block | 1 | 3.189 | 0.074 | – | – | – | |

**(c) Unselected, non-experimentally evolved males: first mating**

| Factor | d.f. | χ^2^ | P | Risk Ratio | Lower CI | | Upper CI |
| --- | --- | --- | --- | --- | --- | --- | --- |
| SPR deficiency | 1 | 3.697 | 0.055 | 1.649 | 0.990 | 2.744 | |
| Block | 1 | 3.470 | 0.063 | – | – | – | |

**(d) Unselected, non-experimentally evolved males: fifth mating**

| Factor | d.f. | χ^2^ | P | Risk Ratio | Lower CI | | Upper CI |
| --- | --- | --- | --- | --- | --- | --- | --- |
| SPR deficiency | 1 | 9.740 | **0.002** | 2.378 | 1.355 | 4.217 | |
| Block | 1 | 1.095 | 0.295 | – | – | – | |

**Table S2. Copulation duration in second matings**

Effects of the *SPR*- deficiency itself in unselected flies, or experimental evolution in *SPR*- and control populations, on copulation duration at a second mating for experimental females paired with wild-type males, or experimental males and females paired within treatment and replicate population. We measured the copulation duration of experimental males paired with wild-type females at first matings only (see main text).

| **Test** | **Male type^1^** | **Female type^1^** | ***SPR*- mean (min) ± S.E.** | **Control mean (min) ± S.E.** | **F** | **df** | **P** |
| --- | --- | --- | --- | --- | --- | --- | --- |
| Effects of *SPR-* deficiency in unselected females | WT | *SPR-* or C | 18.9 ± 1.0 | 19.5 ± 1.0 | 0.2 | 1,70 | 0.67 |
| Experimental evolution of females, generation 26 | WT | *SPR-* or C | 18.7 ± 0.6 | 19.5 ± 0.7 | 0.7 | 1,6.0 | 0.45 |
| Effects of *SPR*- deficiency in unselected within-treatment pairs | *SPR-* or C | *SPR-* or C | 18.2 ± 1.3 | 16.9 ± 1.3 | 0.5 | 1,35 | 0.48 |
| Experimental evolution in within-treatment pairs, generation 26 | *SPR-* or C | *SPR-* or C | 18.6 ± 0.7 | 17.4 ± 0.8 | 1.3 | 1,7.1 | 0.28 |

^1^ Males and females were *SPR*- or genetically matched controls (C), or wild-type (WT)

**Table S3: Post-mating fecundity**

Effects of the *SPR*- deficiency itself in unselected flies, or experimental evolution in *SPR*- and control populations, on post-mating fecundity over 24 hours for (a) wild-type females that mated with experimental males or (b) experimental females that mated with wild-type males

| **Test** | **Male type^1^** | **Female type^1^** | ***SPR*- mean N eggs ± S.E.** | **Control mean N eggs ± S.E.** | **F** | **df** | **P** |
| --- | --- | --- | --- | --- | --- | --- | --- |
| (a) Effects of *SPR*- deficiency in unselected males | *SPR-* or C | WT | 74.5 ± 4.7 | 64.8 ± 5.1 | 1.9 | 1,35 | 0.18 |
| Experimental evolution of males, generation 16 | *SPR-* or C | WT | 63.1 ± 1.2 | 61.7 ± 1.3 | 0.7 | 1,6.1 | 0.43 |
| (b) Effects of *SPR*- deficiency in unselected females | WT | *SPR-* or C | 22 ± 6 | 71 ± 5 | 35.8 | 1,56 | **<0.0001** |
| Experimental evolution of females, generation 26 | WT | *SPR-* or C | 27 ± 5 | 62 ± 5 | 22.1 | 1,6.9 | **0.0023** |

^1^ Males and females were *SPR*- or genetically matched controls (C), or wild-type (WT)

**Table S4.** Full model results for the latency until re-mating of wild-type females after initially mating with (a) experimentally evolved *SPR-* and control males for the males’ first mating, (b) experimentally evolved *SPR-* and control males for the males’ fifth consecutive mating, (c) unselected *SPR-* and control males the males’ first mating, and (d) unselected *SPR-* and control males for the males’ fifth mating

**(a) Experimentally evolved males at generation 36: first mating**

| Factor | d.f. | χ^2^ | P | Risk Ratio | Lower CI | | Upper CI |
| --- | --- | --- | --- | --- | --- | --- | --- |
| Treatment | 1 | 4.169 | **0.041** | 1.364 | 1.012 | 1.845 | |
| SPR deficiency | 1 | 0.356 | 0.551 | 1.094 | 0.812 | 1.472 | |
| Block | 1 | 1.566 | 0.211 | – | – | – | |

**(b) Experimentally evolved males at generation 36: fifth mating (i.e., with a depleted male)**

| Factor | d.f. | χ^2^ | P | Risk Ratio | Lower CI | | Upper CI |
| --- | --- | --- | --- | --- | --- | --- | --- |
| Treatment | 1 | 2.348 | 0.126 | 1.234 | 0.943 | 1.616 | |
| SPR deficiency | 1 | 2.431 | 0.119 | 1.239 | 0.946 | 1.621 | |
| Block | 1 | 1.647 | 0.199 | – | – | – | |

**(c) Unselected, non-experimentally evolved males: first mating**

| Factor | d.f. | χ^2^ | P | Risk Ratio | Lower CI | | Upper CI |
| --- | --- | --- | --- | --- | --- | --- | --- |
| SPR deficiency | 1 | 1.710 | 0.191 | 0.622 | 0.297 | 1.267 | |
| Block | 1 | 7.713 | 0.006 | – | – | – | |

**(d) Unselected, non-experimentally evolved males: fifth mating**

| Factor | d.f. | χ^2^ | P | Risk Ratio | Lower CI | | Upper CI |
| --- | --- | --- | --- | --- | --- | --- | --- |
| SPR deficiency | 1 | 0.182 | 0.670 | 0.884 | 0.502 | 1.568 | |
| Block | 1 | 1.262 | 0.261 | – | – | – | |

**Table S5: Morphological evolution**

Effects of the *SPR*- deficiency itself in unselected flies, or experimental evolution in *SPR*- and control populations, on male and female mass (with females tested only for effects of the deficiency itself, as a check for pleiotropic effects of the deficiency) and male testes and accessory gland size with both absolute and body-mass-adjusted measures

| **Test** | **Trait** | ***SPR*- mean ± S.E.** | **Control mean ± S.E.** | **F** | **df** | **P** |
| --- | --- | --- | --- | --- | --- | --- |
| Effects of *SPR*- deficiency in unselected flies | female mass (mg) | 1.39 ± 0.03 | 1.37 ± 0.03 | 2.4 | 1,32 | 0.13 |
| Effects of *SPR*- deficiency in unselected flies | male mass (mg) | 0.78 ± 0.02 | 0.73 ± 0.02 | 0.3 | 1,30 | 0.62 |
| Experimental evolution of males (generation 16) | male mass (mg) | 0.86 ± 0.01 | 0.83 ± 0.01 | 2.4 | 1,6.0 | 0.17 |
| Effects of *SPR*- deficiency in unselected flies | testes (mm^2^) | 0.205 ± 0.006 | 0.218 ± 0.007 | 2.2 | 1,25 | 0.15 |
|  | accessory gland (mm^2^) | 0.168 ± 0.005 | 0.178 ± 0.006 | 1.5 | 1,25 | 0.23 |
| Experimental evolution of males (generation 16) | testes (mm^2^) | 0.152 ± 0.004 | 0.149 ± 0.005 | 0.1 | 1,6.2 | 0.72 |
|  | accessory gland (mm^2^) | 0.212 ± 0.005 | 0.214 ± 0.005 | 0.1 | 1,6.3 | 0.81 |
|  | body mass-adjusted testes area (mm^2^/mg) | 0.177 ± 0.004 | 0.181 ± 0.004 | 0.3 | 1,6.4 | 0.59 |
|  | body mass-adjusted accessory gland area (mm^2^/mg) | 0.246 ± 0.006 | 0.258 ± 0.005 | 2.3 | 1,6.1 | 0.18 |

**Table S6.** Full model results for the proportion of offspring sired by males when first to mate with a wild-type female (P1), for (a) experimentally evolved *SPR-* and control males for the males’ first mating, (b) experimentally evolved *SPR-* and control males for the males’ fifth consecutive mating, (c) unselected *SPR-* and control males for the males’ first mating, and (d) unselected *SPR-* and control males for the males’ fifth mating

**(a) Experimentally evolved males at generation 36: first mating**

| Factor | t-value | d.f. |  | P |
| --- | --- | --- | --- | --- |
| Treatment | -1.4 | 1,6 |  | 0.111 |
| SPR deficiency | -1.0 | 1,284 |  | 0.31 |
| Remating day | -0.8 | 1,284 |  | 0.41 |
| 1^st^ mating duration | 0.6 | 1,284 |  | 0.57 |
| Remating duration | 4.1 | 1,284 |  | **<0.0001** |

**(b) Experimentally evolved males at generation 36: fifth mating**

| Factor | t-value | d.f. |  | P |
| --- | --- | --- | --- | --- |
| Treatment | -1.5 | 1,6 |  | 0.19 |
| SPR deficiency | -0.7 | 1,224 |  | 0.47 |
| Remating day | 2.3 | 1,224 |  | **0.02** |
| 1^st^ mating duration | 2.1 | 1,224 |  | **0.04** |
| Remating duration | -0.3 | 1,224 |  | **0.80** |

**(c) Unselected, non-experimentally evolved males: first mating**

| Factor | d.f. | Deviance | F | P |
| --- | --- | --- | --- | --- |
| SPR deficiency | 1,47 | 37.01 | 3.277 | 0.077 |
| Remating day | 1,46 | 1.008 | 0.089 | 0.767 |
| 1^st^ mating duration | 1,45 | 10.61 | 0.940 | 0.338 |
| Remating duration | 1,44 | 0.074 | 0.007 | 0.936 |
| Block | 1,43 | 3.431 | 0.304 | 0.584 |

**(d) Unselected, non-experimentally evolved males: fifth mating**

| Factor | d.f. | Deviance | | F | | P | |  |
| --- | --- | --- | --- | --- | --- | --- | --- | --- |
| SPR deficiency | 1,36 | | 0.338 | | 0.051 | | 0.823 | |
| Remating day | 1,35 | | 1.173 | | 0.176 | | 0.678 | |
| 1^st^ mating duration | 1,34 | | 4.330 | | 0.650 | | 0.426 | |
| Remating duration | 1,33 | | 1.198 | | 0.180 | | 0.674 | |
| Block | 1,32 | | 3.142 | | 0.472 | | 0.497 | |

**Table S7.** Full model results for the proportion of offspring sired by males when second to mate with a wild-type female (P2), for (a) experimentally evolved males and (b) unselected males with and without the SPR deficiency

**(a) Experimentally evolved males at generation 36**

| Factor | t-value | d.f. |  | P |
| --- | --- | --- | --- | --- |
| Treatment | 2.2 | 1,6 |  | 0.07 |
| SPR deficiency | 2.5 | 1,221 |  | **0.01** |
| 1^st^ mating duration | -0.2 | 1,221 |  | 0.83 |
| Remating duration | -3.4 | 1,221 |  | **0.001** |

**(b) Unselected, non-experimentally evolved males**

| Factor | d.f. | Deviance | F | P |
| --- | --- | --- | --- | --- |
| SPR deficiency | 1,35 | 3.474 | 0.242 | 0.626 |
| 1^st^ mating duration | 1,34 | 0.105 | 0.007 | 0.932 |
| Remating duration | 1,33 | 5.643 | 0.394 | 0.535 |

**Table S8.** Characteristics of calibration curves for *SP*, *Dup99B* and *RpL32* (mean across 8 plates)

|  | *SP* (*Acp70A*) | *Dup99B* | *RpL32* |
| --- | --- | --- | --- |
| Mean slope | -3.064 | -3.21 | -3.51 |
| Mean intercept | 54.83 | 61.25 | 66.14 |
| Mean efficiency % | 112.05 | 105 | 92.84 |
| Mean r^2^ | 0.983 | 0.987 | 0.98 |

**Table S9.** Primer characteristics for the target genes (*sex peptide* [*SP*; *Acp70A*] and *Dup99B*) and reference gene (*RpL32*)

|  | ***SP* (*Acp70A*)** | ***Dup99B*** | ***RpL32*** |
| --- | --- | --- | --- |
| **NCBI accession for mRNA** | NM_079333.2 | NM_206582.2 | NM_079843.4, NM_170461.3, NM_170460.2, NM_001144655.3 |
| **Forward primer sequence** | TGGGAAT GGCCGTG GAATAG | CAGAAGGA TCGTGAGA AGTGGTGC | TGCTAAGC TGTCGCA CAAATGG |
| **Reverse primer sequence** | CGGCACCA CTTATCA CGAGG | TTCGGCAT CTGCCAC CGAGGTA | TGCGCTTG TTCGATC CGTAAC |
| **Location of primer** | Reverse primer resides on two exons | Forward primer resides on two exons | Forward primer resides on two exons |
| **Specificity screen (BLAST)** | The primers may not be specific to the input template. However, the product on potentially unintended templates was much larger (681 bp; NM_001104369.2 *Drosophila melanogaster* Inositol-requiring enzyme-1 (Ire1), mRNA). | The primers were specific to the input template | The primers were specific to the input template. |
| **Amplicon length** | 74 bp | 64 bp | 113 bp |
| **Splice variants targeted** | No splice variants | Of the 2 splice variants, the primers targeted transcript variant A. | All four splice variants A, B, C and D |

# Supplementary figure legends

**Figure S1. Backcrossing to generate unselected SPR- and control starting populations.**

The SPR- deficiency (*Df(1)Exel6234*) was serially backcrossed into the white^Dahomey^ genetic background. Following 5 generations of crosses, the offspring were crossed together and SPR- homozygote and control populations were established. Final generation Ne = 300.

**Figure S2. Backcrossing to generate evolved control lines carrying the SPR- deletion**

The SPR- deficiency (*Df(1)Exel6234*) was serially backcrossed into evolved control populations to generate lines with the control genetic backgound but which posses red-eyes and carry the SPR- deficiency.

**Figure S3. Backcrossing to generate lines that evolved under high promiscuity (SPR-) form which the SPR- deletion has been removed**

The wild-type SPR was serially backcrossed into evolved SPR- populations to generate lines with a genetic background that evolved with SPR- and thus high promiscuity, but which posses white eyes and a wild-type SPR gene (i.e. they lack the *Df(1)Exel6234* deficiency).
