# Supplementary material for: Experimental evolution under hyper-promiscuity in Drosophila melanogaster
Source: BMC Evol Biol. 2016 Jun 16;16:131. doi: 10.1186/s12862-016-0699-8 (PMC4910217; doi:10.1186/s12862-016-0699-8)
Supplement: Additional file 2: Figure S1. — The backcrossing procedure used to generate the experimental populations. Figure S2. The backcrossing procedure used to generate SPR+ evolved controls for the sperm competition experiments. Figure S3. The backcrossing procedure used to generate SPR- evolved controls for the sperm competition experiments. (PPT 368 kb) [file 12862_2016_699_MOESM2_ESM.ppt]

## Slide 1
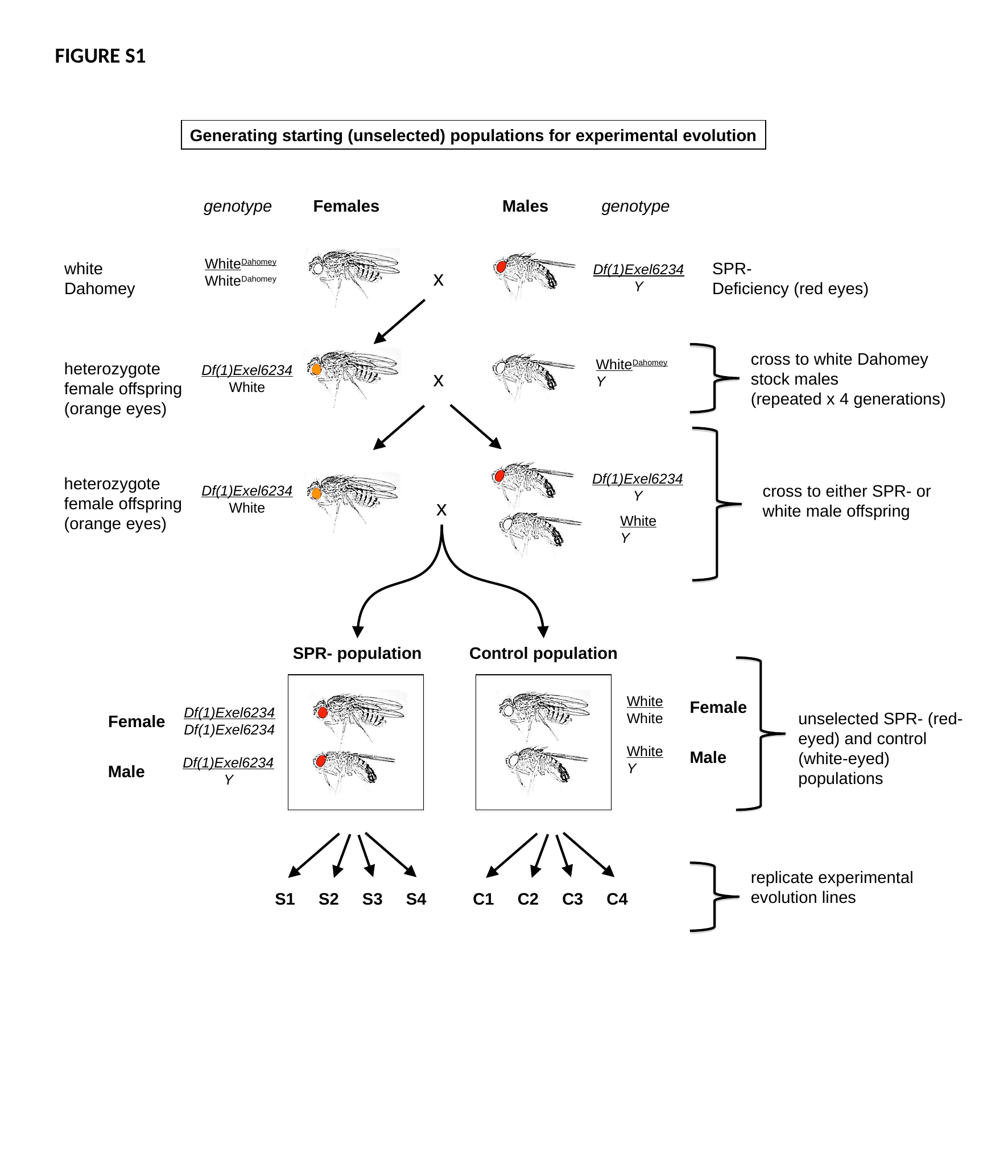

FIGURE S1
Generating starting (unselected) populations for experimental evolution
genotype
Females
Males
genotype
WhiteDahomey
WhiteDahomey
white
Dahomey
SPR-
Deficiency (red eyes)
Df(1)Exel6234
Y
x
cross to white Dahomey
stock males
(repeated x 4 generations)
WhiteDahomey
Y
heterozygote
female offspring
(orange eyes)
Df(1)Exel6234
White
x
Df(1)Exel6234
Y
heterozygote
female offspring
(orange eyes)
cross to either SPR- or white male offspring
Df(1)Exel6234
White
x
White
Y
SPR- population
Control population
White
White
Female
Df(1)Exel6234
Df(1)Exel6234
unselected SPR- (red-eyed) and control (white-eyed) populations
Female
White
Y
Male
Df(1)Exel6234
Y
Male
replicate experimental evolution lines
 S1 S2 S3 S4
 C1 C2 C3 C4

## Slide 2
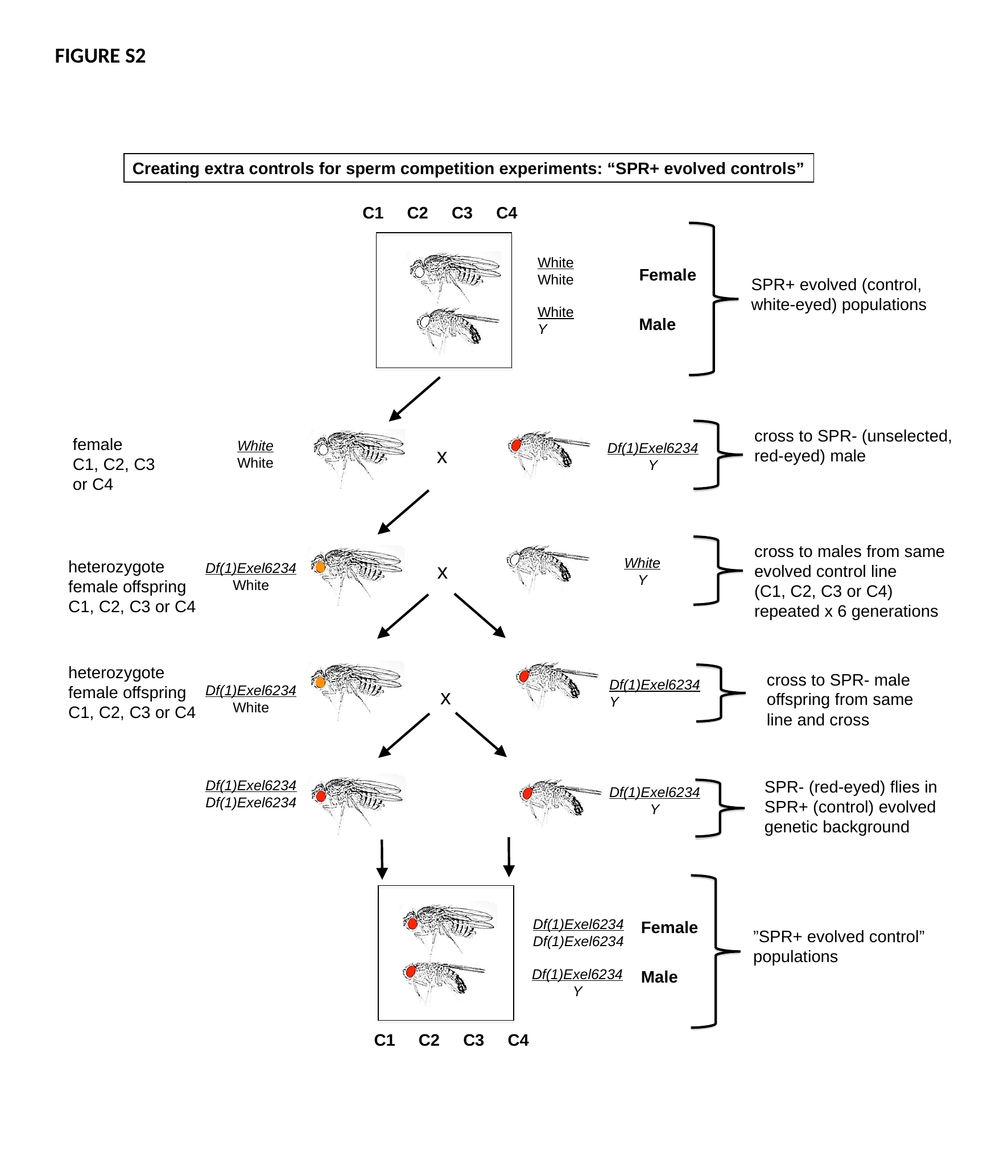

FIGURE S2
Creating extra controls for sperm competition experiments: “SPR+ evolved controls”
 C1 C2 C3 C4
White
White
Female
SPR+ evolved (control, white-eyed) populations
White
Y
Male
cross to SPR- (unselected, red-eyed) male
female
C1, C2, C3
or C4
White
White
Df(1)Exel6234
Y
x
cross to males from same evolved control line
(C1, C2, C3 or C4)
repeated x 6 generations
White
Y
heterozygote
female offspring
C1, C2, C3 or C4
x
Df(1)Exel6234
White
heterozygote
female offspring
C1, C2, C3 or C4
cross to SPR- male offspring from same line and cross
Df(1)Exel6234
Y
Df(1)Exel6234
White
x
Df(1)Exel6234
Df(1)Exel6234
SPR- (red-eyed) flies in SPR+ (control) evolved genetic background
Df(1)Exel6234
Y
Df(1)Exel6234
Df(1)Exel6234
Female
”SPR+ evolved control” populations
Df(1)Exel6234
Y
Male
 C1 C2 C3 C4

## Slide 3
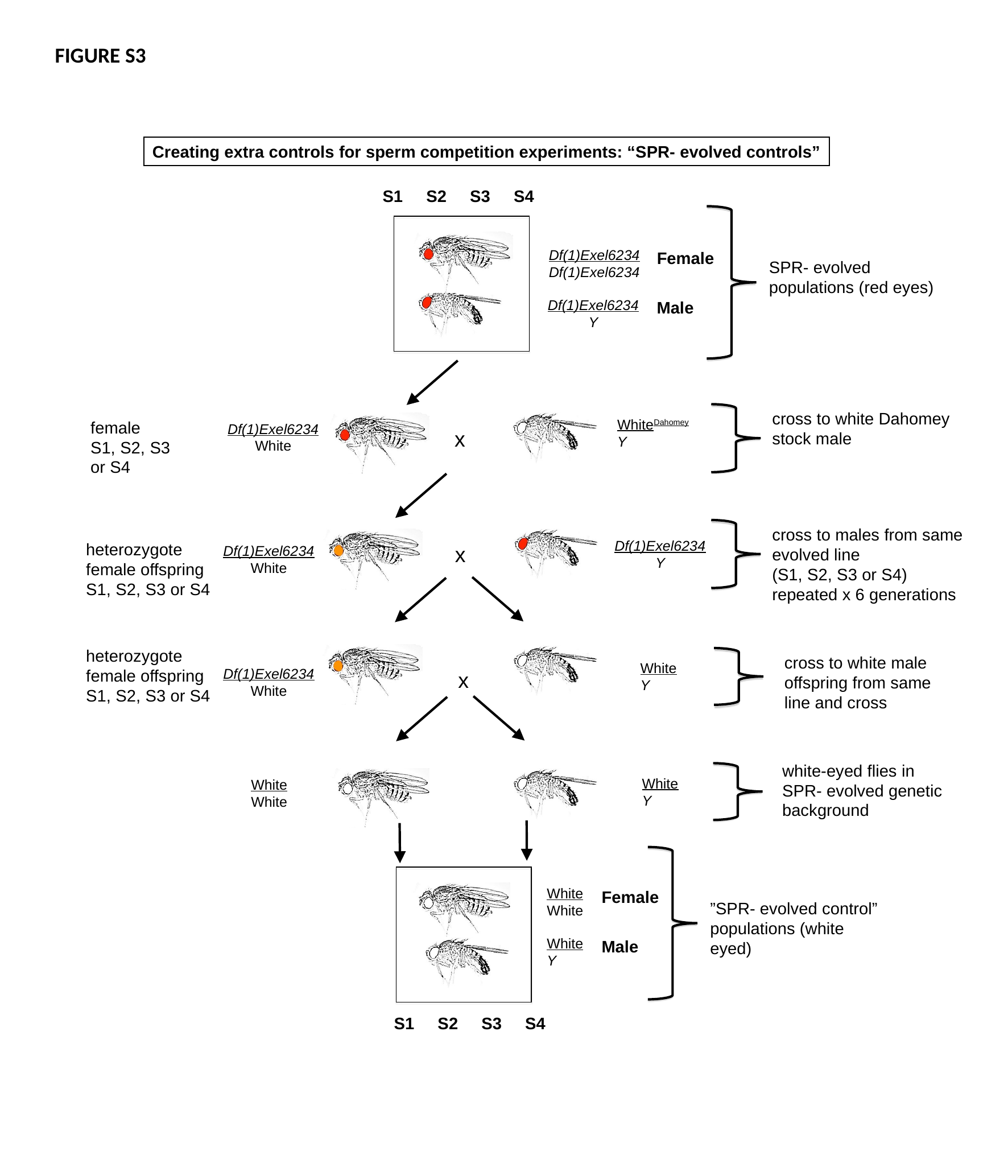

FIGURE S3
Creating extra controls for sperm competition experiments: “SPR- evolved controls”
 S1 S2 S3 S4
Df(1)Exel6234
Df(1)Exel6234
Female
SPR- evolved populations (red eyes)
Df(1)Exel6234
Y
Male
cross to white Dahomey
stock male
WhiteDahomey
Y
female
S1, S2, S3
or S4
Df(1)Exel6234
White
x
cross to males from same evolved line
(S1, S2, S3 or S4)
repeated x 6 generations
Df(1)Exel6234
Y
heterozygote
female offspring
S1, S2, S3 or S4
x
Df(1)Exel6234
White
heterozygote
female offspring
S1, S2, S3 or S4
cross to white male offspring from same line and cross
White
Y
Df(1)Exel6234
White
x
white-eyed flies in
SPR- evolved genetic background
White
Y
White
White
White
White
Female
”SPR- evolved control” populations (white eyed)
White
Y
Male
 S1 S2 S3 S4
